# On some Cuban species of the genus *Longior* Travassos & Kloss, 1958 (Oxyurida, Hystrignathidae),  with description of a new species

**DOI:** 10.3897/zookeys.78.958

**Published:** 2011-01-28

**Authors:** Jans Morffe, Nayla García

**Affiliations:** Instituto de Ecología y Sistemática, Instituto de Ecología y Sistemática, Carretera de Varona km 31/2, Capdevila, Boyeros, A.P. 8029, C.P. 10800, Havana, Cuba

**Keywords:** Nematoda, Oxyurida, Hystrignathidae, Passalidae, *Longior*, *Passalus*, Cuba, new species

## Abstract

Longior zayasi Coy, García & Alvarez, 1993 is established as *incertae sedis* because the males (declared as the holotype) are inconsistent with the generic diagnosis, particularly in relation to the morphology of the head and tail. Thus, the females of Longior zayasi species (which agree with the generic diagnosis) are renamed and re-described as Longior longior Morffe & García **sp. n.** We also described males found in the sample and considered as conspecific with the new species. A comparative table with the measurements of the most of the records of Longior longior is given. The male of Longior similis Morffe, García & Ventosa, 2009 is described from the type locality of the species and compared with the known males of the genus. A key to the females of the Cuban Longior is given.

## Introduction

The genus Longior Travassos & Kloss, 1958, includes six nominal species of monoxenous parasites from passalid beetles. All of these species have been described from the Neotropical region: Brazil and the West Indies (Saint Lucia and Cuba). The genus is distinguished by having females with the unarmed cervical cuticle, sub-cylindrical procorpus, a monodelphic-prodelphic genital tract and longitudinally ridged eggs (Adamson and Van Waerebeke 1992).

At present, the male is known only in two species: Longior longicollis Travassos & Kloss, 1958 (Brazil) and Longior zayasi Coy, García & Alvarez, 1993 (Cuba) (Travassos and Kloss 1958, Coy et al. 1993). However, the latter shows marked differences in the morphology of the cephalic and tail ends with regard to Longior longicollis, to which the first male described in the genus belongs. These differences make necessary the analysis and discussion of the taxonomical status of Longior zayasi. Several authors (Van Waerebeke 1973; Hunt 1981) have referred to the problems involved in assigning a male to its correct species in cases of multiple co-infection with multiple species of parasites. They claimed as their main reasons the lack of knowledge about the generic features of the males in most genera within the family (the male is known only in a few genera) and their morphological homogeneity. For these reasons misidentifications can be made in some cases.

In this work, we discuss the taxonomical status of Longior zayasi, describe a new species of the genus and describe the male of Longior similis Morffe, García &Ventosa, 2009.

## Material and methods

All the passalids for this study were collected by hand from rotting logs in Cuba. Two specimens of Passalus pertyi were caught from La Jaula, La Habana Province and eight specimens of Passalus interstitialis from Escaleras de Jaruco, La Habana Province. Beetles were kept alive in plastic jars with wood chips as food. They were killed with ethyl ether and processed following Morffe et al. (2009). Intestines were dissected in normal saline and nematodes were removed from host guts and killed with hot water (60–80ºC). They were fixed in 70% ethanol and clear-mounted in glycerine. The edges of the coverslips were sealed with nail polish.

Two additional specimens of Passalus pertyi from La Melba, Holguín Province, Cuba were included in the present study in order to obtain males of Longior longior sp. n. Hosts were killed and conserved in 70% ethanol. Dissection of the beetles and processing of the parasites were made as described above, but using water instead of normal saline.

Measurements of the nematodes were made with a calibrated eyepiece micrometer and are given in millimeters. De Man’s ratios a, b, c and V% were calculated. Micrographs were taken with an AxioCam digital camera attached to a Carl Zeiss AxioScop 2 Plus compound microscope. Line drawings were made on the basis of micrographs using the softwares CorelDRAW X3 and Adobe Photoshop CS2. Scale bars of all Figures are given in millimeters.

The material examined is deposited in the Colección Helmintológica de las Colecciones Zoológicas (CZACC), Instituto de Ecología y Sistemática, Havana, Cuba.

## Systematics

### Genus Longior

Travassos & Kloss, 1958

Figure 1 A–D

Longior zayasi Coy, García & Alvarez, 1993 *incertae sedis*

#### General.

Coy et al. (1993) described Longior zayasi, from El Salón, Sierra del Rosario, eastern Pinar del Río Province, Cuba. Althought the females morphology is consistent with the generic definition, examination of the male specimens showed features that disagree with the diagnosis of the male of Longior longicollis,the type species of the genus. These features are:

First cephalic annule notably long, surpassing the stoma length, inflated, forming a truncated cone-like structure. According with Travassos and Kloss (1958), Longior longicollis has a cephalic dilatation hardly conspicuous.

Posterior end finished in a sharply pointed tail, instead of the ventral expansion of the tail cuticle in Longior longicollis that forms a bursa-like structure, referred by the authors as a “ventral valve”.

These discrepancies support the possibility that the males attributed to Longior zayasi are misplaced in this genus. So far, we can not assign these males to a genus, although is possible that they belong to some species of Artigasia Christie, 1934 or Hystrignathus Leidy, 1850 where the females show a long and dilated first cephalic annule. New collections from the locality are needed to clarify the status of Longior zayasi by comparing males with the females in the same host. Meanwhile we consider this species as an *incertae sedis*.

Coy et al. (1993) established a male specimen as holotype of Longior zayasi. According to the Article 73.1 of the International Code of Zoological Nomenclature “A holotype is the single specimen upon which a new nominal species-group taxon is based in the original publication…”. As a new name becomes necessary for the females described as conspecific with Longior zayasi, below we re-describe and rename such females, including the description of sympatric males that present the diagnostic features of Longior and we consider as conspecific with those females.

**Figure 1.**
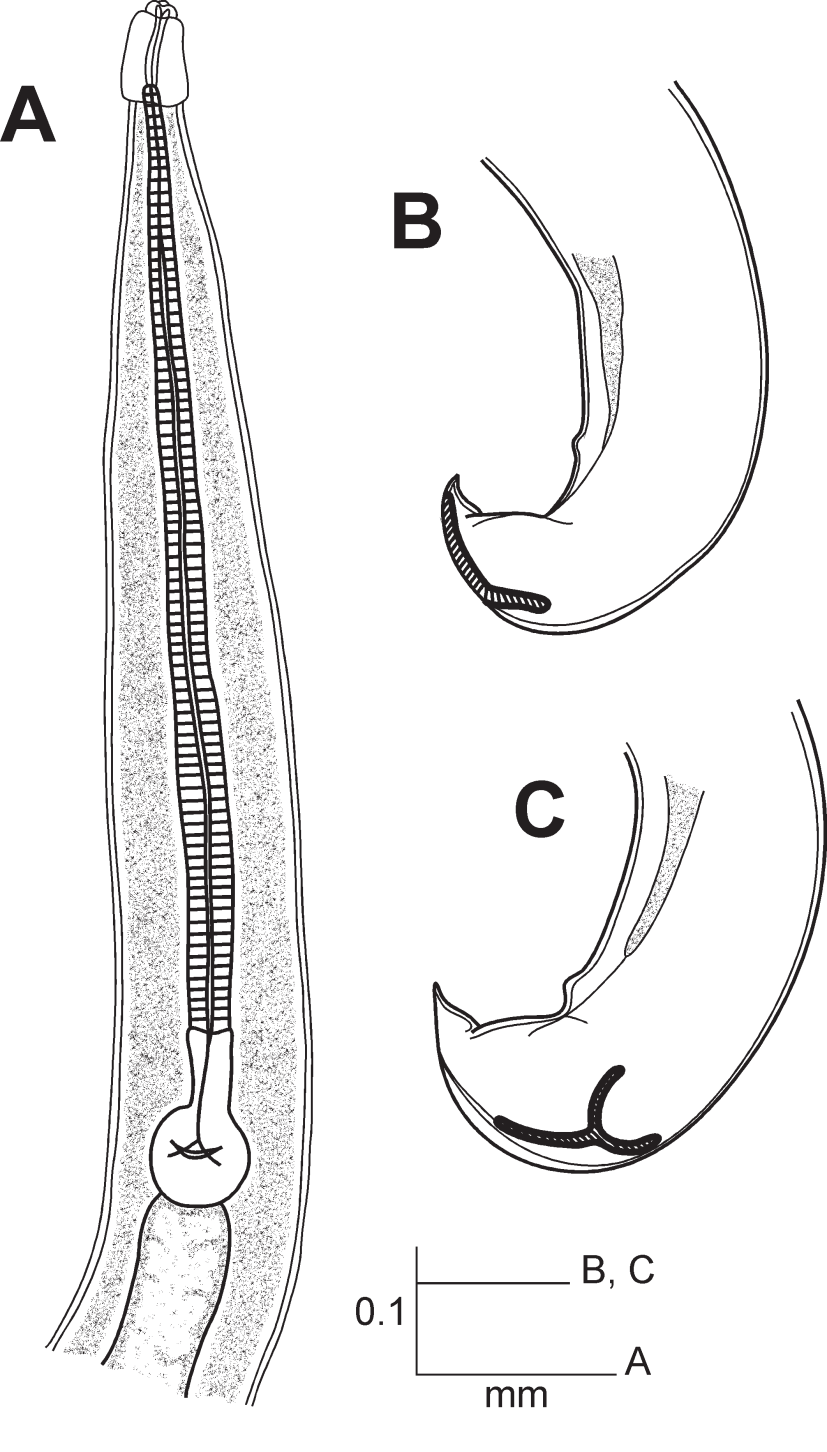
Longior zayasi Coy, García & Alvarez, 1993; *incertae sedis* (male). Drawing modified from Coy et al. (1993). **A** Esophageal region **B** Tail, lateral view **C** Tail, dorso-lateral view.

### Longior longior sp. n.

urn:lsid:zoobank.org:act:20168FE9-8444-42C2-A9DD-F3E8D423A232

**Figure 2.**
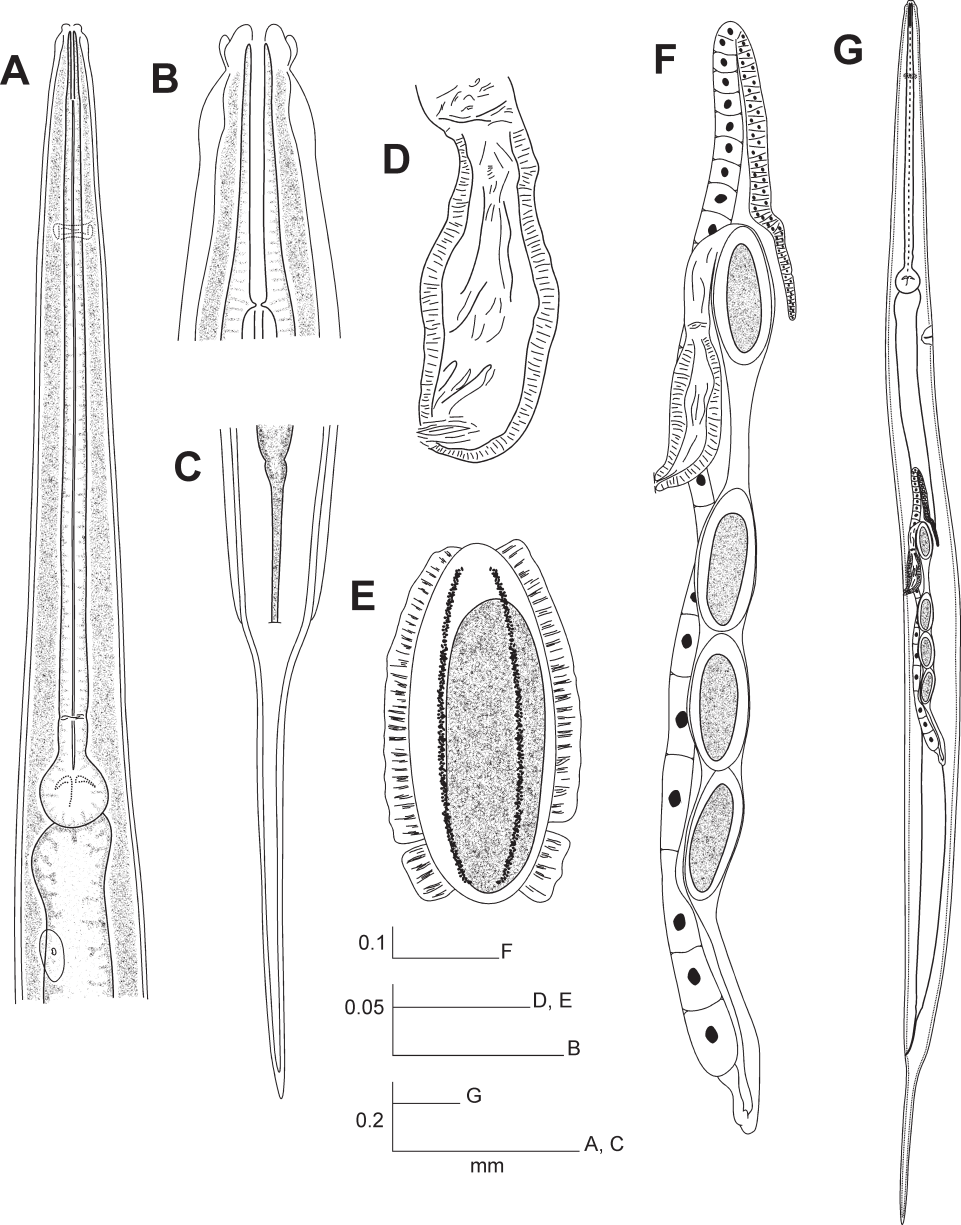
Longior longior Morffe & García sp. n. (female). **A** Esophageal region **B** Cephalic end **C** Tail, ventral view **D** Vulva **E** Egg **F** Genital tract **G** Entire nematode, lateral view.

**Figure 3.**
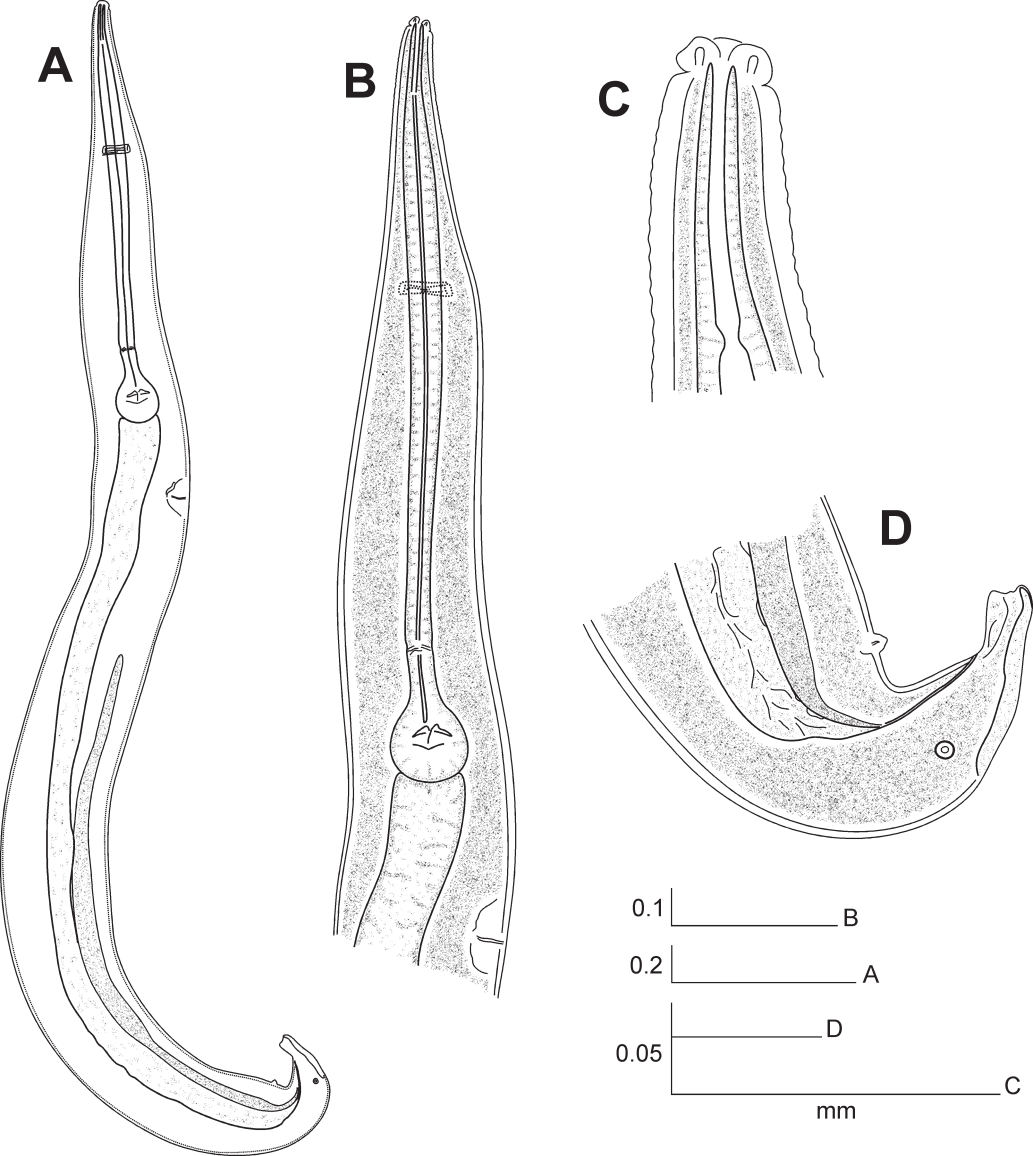
Longior longior Morffe & Garcíasp. n. (male). **A** Entire nematode, lateral view **B** Esophageal region, lateral view **C** Cephalic end **D** Tail, lateral view.

Figure 2 A–G Figure 3 A–D Figure 5 A–F 

#### Type material.

♀ holotype; Cuba, La Habana Province, San José de Las Lajas, La Jaula; in Passalus pertyi; 15.III.2008; E. Fonseca, J. Morffe, F. Alvarez coll.; CZACC 11.4487. ♂ allotype, same data as holotype, CZACC 11.4620. 11 adult ♀♀ paratypes, same data as holotype, CZACC 11.4488–11.4498. 5 immature ♀♀ paratypes, CZACC 11.4499–11.4503, same data as holotype. 5 ♂♂ paratypes, same data as holotype, CZACC 11.4621–11.4625.

#### Other materials examined.

4 ♂♂ vouchers; Cuba, Holguín Province, Nipe-Sagua-Baracoa, La Melba; in Passalus pertyi; V.2007; R. Barba, D. Ortiz coll.; CZACC 11.4626–11.4629.

3 ♀♀ paratypes of Longior zayasi; Cuba, Pinar del Río Province, Sierra del Rosario, El Salón; in Passalus interstitialis; 1989; A. Coy, M. Alvarez coll.; CZACC 11.4178–11.4180.

#### Measurements.

Holotype (female) a = 23.78, b = 4.63, c = 7.04, V% = 51.70, total length = 3.520, maximum body width = 0.148, first cephalic annule (length×width) = 0.028×0.035, stoma length = 0.078, procorpus length = 0.640, isthmus length = 0.045, diameter of basal bulb = 0.070, total length of oesophagus = 0.760, nerve ring to anterior end = 0.233, excretory pore to anterior end = 0.980, vulva to posterior end = 1.700, anus to posterior end = 0.500, eggs = 0.123–0.128×0.045–0.053 (0.125 ± 0.003×0.049 ± 0.004 n = 3).

Female adult paratypes (n = 11) a = 23.61–28.28 (25.00 ± 1.57 n = 11), b = 4.49–5.45 (4.81 ± 0.29 n = 10), c = 6.24–8.54 (7.37 ± 0.66 n = 11), V% = 49.73–58.01 (52.98 ± 2.34 n = 11), total length = 3.500–4.525 (3.863 ± 0.277 n = 11), maximum body width = 0.138–0.173 (0.155 ± 0.010 n = 11), first cephalic annule (length×width) = 0.023–0.028×0.035–0.038 (0.024 ± 0.002×0.036 ± 0.001 n = 9), stoma length = 0.073–0.085 (0.079 ± 0.005 n = 11), procorpus length = 0.630–0.720 (0.678 ± 0.027 n = 10), isthmus length = 0.040–0.058 (0.053 ± 0.005 n = 10), diameter of basal bulb = 0.065–0.078 (0.071 ± 0.004 n = 10), total length of oesophagus = 0.760–0.860 (0.806 ± 0.030 n = 10), nerve ring to anterior end = 0.230–0.250 (0.242 ± 0.007 n = 9), excretory pore to anterior end = 0.970–1.125 (1.050 ± 0.047 n = 10), vulva to posterior end = 1.600–1.925 (1.813 ± 0.097 n = 11), anus to posterior end = 0.470–0.590 (0.526 ± 0.038 n = 11), eggs = 0.110–0.143×0.050–0.060 (0.133 ± 0.006×0.053 ± 0.003 n = 28).

Female immature paratypes (n = 5) a = 24.31–27.02 (24.96 ± 1.16 n = 5), b = 4.13–4.42 (4.28 ± 0.12 n = 5), c = 7.07–8.86 (7.74 ± 0.68 n = 5), V% = 53.54–56.56 (54.99 ± 1.07 n = 5), total length = 3.050–3.325 (3.165 ± 0.104 n = 5), maximum body width = 0.118–0.135 (0.127 ± 0.006 n = 5), first cephalic annule (length×width) = 0.023–0.025×0.035 (0.024 ± 0.001×0.035 ± 0.000 n = 5), stoma length = 0.070–0.073 (0.072 ± 0.001 n = 5), procorpus length = 0.610–0.680 (0.638 ± 0.028 n = 5), isthmus length = 0.055–0.058 (0.056 ± 0.001 n = 5), diameter of basal bulb = 0.063–0.068 (0.065 ± 0.002 n = 5), total length of oesophagus = 0.690–0.760 (0.740 ± 0.029 n = 5), nerve ring to anterior end = 0.223–0.233 (0.227 ± 0.004 n = 5), excretory pore to anterior end = 0.910–0.980 (0.942 ± 0.031 n = 5), vulva to posterior end = 1.325–1.500 (1.425 ± 0.068 n = 5), anus to posterior end = 0.350–0.470 (0.412 ± 0.045 n = 5).

Allotype (male) a = 15.68, b = 3.63, c = 49.67, total length = 1.490, maximum body width = 0.095, stoma length = 0.038, procorpus length = 0.330, isthmus length = 0.033, diameter of basal bulb = 0.045, total length of oesophagus = 0.410, nerve ring to anterior end = 0.160, excretory pore to anterior end = 0.560, cloaca to posterior end = 0.030, distance from the mammiform papillae to the posterior end = 0.130, distance from the dorso-lateral papillae to the posterior end = 0.065.

Male paratypes (n = 5) a = 11.56–14.10 (12.40 ± 1.04 n = 5), b = 3.13–3.60 (3.38 ± 0.17 n = 5), c = 43.33–51.27 (47.13 ± 3.54 n = 5), total length = 1.220–1.510 (1.366 ± 0.111 n = 5), maximum body width = 0.100–0.120 (0.111 ± 0.010 n = 5), stoma length = 0.040–0.048 (0.043 ± 0.003 n = 5), procorpus length = 0.300–0.340 (0.322 ± 0.018 n = 5), isthmus length = 0.030–0.033 (0.031 ± 0.001 n = 5), diameter of basal bulb = 0.045–0.048 (0.047 ± 0.001 n = 5), total length of oesophagus = 0.390–0.420 (0.404 ± 0.015 n = 5), nerve ring to anterior end = 0.150–0.168 (0.159 ± 0.007 n = 5), excretory pore to anterior end = 0.480–0.550 (0.512 ± 0.028 n = 5), cloaca to posterior end = 0.028–0.030 (0.029 ± 0.001 n = 5), distance from the mammiform papillae to the posterior end = 0.128–0.148 (0.137 ± 0.010 n = 3), distance from the dorso-lateral papillae to the posterior end = 0.058–0.065 (0.062 ± 0.003 n = 5).

#### Population from La Melba, Nipe-Sagua_Baracoa, Holguín Province.

Males (n = 4) a = 11.55–14.00 (13.00 ± 1.12 n = 4), b = 3.10–4.20 (3.50 ± 0.48 n = 4), c = 33.87–61.09 (44.96 ± 11.49 n = 4), total length = 1.170–1.680 (1.348 ± 0.227 n = 4), maximum body width = 0.085–0.120 (0.104 ± 0.015 n = 4), stoma length = 0.038–0.040 (0.039 ± 0.001 n = 4), procorpus length = 0.283–0.320 (0.303 ± 0.016 n = 4), isthmus length = 0.028–0.038 (0.032 ± 0.004 n = 4), diameter of basal bulb = 0.050–0.063 (0.054 ± 0.006 n = 4), total length of oesophagus = 0.378–0.400 (0.384 ± 0.010 n = 4), nerve ring to anterior end = 0.133–0.158 (0.145 ± 0.013 n = 3), excretory pore to anterior end = 0.430–0.490 (0.463 ± 0.025 n = 4), cloaca to posterior end = 0.028–0.038 (0.031 ± 0.005 n = 4), distance from the mammiform papillae to the posterior end = 0.115–0.133 (0.125 ± 0.009 n = 3), distance from the dorso-lateral papillae to the posterior end = 0.055–0.070 (0.063 ± 0.006 n = 4).

#### Population from El Salón, Sierra del Rosario, Pinar del Río Province.

Females (n = 3) a = 17.20–23.94 (21.67 ± 3.87), b = 4.79–4.86 (4.83 ± 0.05), c = 6.68–7.63 (7.25 ± 0.51), V% = 45.99–52.19 (50.04 ± 3.51), total length = 3.580–3.890 (3.737 ± 0.155), maximum body width = 0.150–0.218 (0.177 ± 0.036), first cephalic annule = 0.018–0.025×0.035–0.038 (0.021 ± 0.005×0.036 ± 0.002), stoma length = 0.060–0.075 (0.066 ± 0.008), procorpus length = 0.650–0.660 (0.655 ± 0.007), isthmus length = 0.050–0.058 (0.053 ± 0.004), diameter of basal bulb = 0.070–0.073 (0.071 ± 0.002), total length of oesophagus = 0.780–0.800 (0.790 ± 0.014), nerve ring to anterior end = 0.225–0.235 (0.230 ± 0.005), excretory pore to anterior end = 0.990–1.05 (1.020 ± 0.042), vulva to posterior end = 1.720–2.020 (1.867 ± 0.150), anus to posterior end = 0.480–0.560 (0.517 ± 0.040), eggs = 0.128–0.140×0.045–0.060 (0.133 ± 0.005×0.053 ± 0.006 n = 5).

#### Description.

Female. Body long and relatively slender. Cervical cuticle without spines. Subcuticular longitudinal striae present. Lateral alae well developed, arising from about two body widths posterior to the basal bulb up to the level of the anus. Head rounded, set-off from body by a single, deep groove, bearing eight prominent, paired papillae. First cephalic annule slightly inflated, about 2 to 2.5 head-lengths long. Stoma long, slender, about 3.5 first cephalic annule lengths long, surrounded by an esophageal collar. Oesophagus consisting of a very elongated, subcylindrical, muscular procorpus less set-off from the isthmus. Basal bulb sub-spherical, valve plate well developed. Intestine simple, sub-rectilinear, its fore region slightly inflated. Rectum short, anus not prominent. Nerve ring encircling procorpus at about 20% of its length. Excretory pore located at about a body width posterior to basal bulb. Vulva a median transverse slit, near midbody, lips not prominent. Vagina muscular, strongly developed, forwardly directed. Genital tract monodelphic-prodelphic. Ovary reflected at about two body widths posterior to bulb, distal flexure about two body widths long. Eggs ovoid, with eight, rough, very prominent, longitudinal ridges not reaching the poles. Oocytes in a single row. Tail conical, subulate, ending in a fine tip.

Male. Body comparatively stout, shorter than female. Posterior region of body ventrally curved. Cervical cuticle unarmed, visibly annulated up to a little distance before the nerve ring. Sub-cuticular longitudinal striae present. Head set off from body by a single groove and bearing eight small, paired papillae. First cephalic annule not conspicuous. Stoma long, surrounded by an esophageal collar. Oesophagus slightly sub-fusiform, diminishing its diameter toward its second half until equaling the diameter of the isthmus. Basal bulb rounded, valve plate developed. Nerve ring encircling procorpus at about 35% of its length. Excretory pore situated at little more than a body-width posterior to basal bulb. Testis single, full of spermatids, commencing at about 1.5 body-widths posterior to bulb. Spicule absent. A large, median, mammiform precloacal papillae situated at a little less than a body width before the posterior end. A pair of dorso-lateral precloacal papillae located at about 0.5 body-widths before the tail tip. Dorsal cuticle of posterior end thickened from level of the latter pair of papillae to the tail tip. There is a cuticular bursa-like structure in the tail end, almost reaching the level of the tail tip.

#### Differential diagnosis.

Longior longior sp. n. differs from Longior longicollis, from Brazil,by having females with the body larger (3.500–4.525 *vs.* 2.98–3.64) and the tail comparatively longer (c = 6.72–8.54 *vs.* 9.03–9.33). The vulva is slightly displaced forward in the present species (V% = 50.00–58.01 *vs.* 57.72–61.81). The tail of the male of Longior longior sp. n. is comparatively shorter (c = 43.33–51.27: 30.66–32.83), and has the tip unreflexed. In Longior longior the nerve ring is displaced to the first half of the procorpus in both sexes, instead of Longior longicollis sp. n. where this structure is situated in the midpoint of the procorpus.

Longior longior sp. n. differs from Longior similis by lacking wide and marked annule in the cervical cuticle of females. Its body is longer (total length = 3.500–4.525 *vs.* 2.675–3.075) and the first cephalic annule is more inflated. The ovary is reflexed at about two body widths beyond the excretory pore, more posterior than Longior similis where it is reflexed at little less than a body width beyond the excretory pore. Males of the new species differ from Longior similis by having the cervical cuticle markedly annulated instead of Longior similis that presents the cervical cuticle smooth.

Longior longior sp. n. is distinguished from Longior alius García & Coy, 1994 (from Cuba) by having the isthmus shorter and not bent, instead of the isthmus very long and bent of the latter species. From Longior semialata Hunt, 1981, from Saint Lucia, West Indies, differs by the lateral alae that commence before the level of the vulva, the body longer (total length = 3.500–4.525 *vs.* 2.26–2.63) and the oesophagus comparatively shorter (b = 4.49–5.45 *vs.* 3.30–3.70). The lateral alae of Longior semialata start at about the middle of the distance between the vulva and the anus.

Longior longior sp. n. can be differentiated from Longior elieri García, Ventosa & Morffe, 2009 (from Cuba) by having an inflated first cephalic annule and by lacking of wide annule in the cervical region. Longior elieri has a not inflated first cephalic annule and the first third of the cervical cuticle widely annulated.

#### Etymology.

Named after the Latin *longior*: the largest, being this species the largest in the genus.

#### Type host.

Passalus pertyi (Coleoptera, Passalidae)

#### Other host.

Passalus interstitialis Escholtz, 1829 (Coleoptera, Passalidae)

#### Site.

Gut caeca

#### Type locality.

La Jaula, San José de Las Lajas, La Habana Province, Cuba.

#### Other records

*(formerly referred as L. zayasi).* El Salón, Sierra del Rosario, Pinar del Río Province, Cuba (Coy et al. 1993); El Mulo, Sierra del Rosario, Pinar del Río Province, Cuba (García et al. 2009a); La Melba, Nipe-Sagua-Baracoa, Holguín Province, Cuba (García et al. 2009b); La Platica, Sierra Maestra, Granma Province, Cuba (Morffe et al. 2009).

#### Remarks.

All the records of females of Longior longior sp. n. were formerly referred as Longior zayasi and measures of most of them have been published. In order to compare the meristic features between localities a chart with such variables is offered (Table 1).

**Table 1.** Comparative measurements of the females and males of Longior longior Morffe & García sp. n. from its type locality La Jaula, San José de las Lajas, La Habana Province, Cuba and the records from El Salón, Sierra del Rosario, Pinar del Río Province, Cuba; La Melba, Nipe-Sagua-Baracoa, Holguín Province, Cuba and La Platica, Sierra Maestra, Granma Province, Cuba. Measurements are given in milimetres.

*Host*	Passalus pertyi	Passalus interstitialis	Passalus pertyi	Passalus pertyi
*Locality*	La Jaula, La Habana Province (type locality) (n = 12)	El Salón, Pinar del Río Province (n = 3)	La Melba, Holguín Province (n = 6)	La Platica, Granma Province (n = 6)
*Female measurements*				
Total length	3.500–4.525	3.580–3.890	3,390–4,090	3,620–4,150
Body width	0.138–0.173	0.150–0.218	0,150–0,170	0,100–0,140
Stoma length	0.073–0.085	0.060–0.075	0,055–0,060	0,058–0,070
Procorpus length	0.630–0.720	0.650–0.660	0,640–0,740	0,600–0,690
Isthmus length	0.040–0.058	0.050–0.058	0,050–0,063	0,048–0,060
Diameter basal bulb	0.065–0.078	0.070–0.073	0,073–0,080	0,060–0,073
Esophagus length	0.760–0.860	0.780–0.800	0,770–0,950	0,730–0,820
Nerve ring-head end	0.230–0.250	0.225–0.235	0,225–0,250	0,235–0,250
Excretory pore-head end	0.970–1.125	0.990–1.05	0,950–1,040	-
Vulva-tail end	1.600–1.925	1.720–2.020	1,720–2,040	2,000
Anus-tail end	0.470–0.590	0.480–0.560	0,480–0,610	0,600–0,640
Eggs	0.110–0.143×0.050–0.060	0.128–0.140×0.045–0.060	0,135–0,153×0,050–0,070	0,125–0,140×0,050–0,058
a	23.61–28.28	17.20–23.94	21,19–27,27	29,36–36,20
b	4.49–5.45	4.79–4.86	4,27–5,13	4,83–5,41
c	6.24–8.54	6.68–7.63	6,70–7,90	6,03–6,92
V%	49.73–58.01	45.99–52.19	45,65–55,67	49,24
*Male measurements*				
Total length	1.220–1.510	-	1.170–1.680	-
Body width	0.095–0.120	-	0.085–0.120	-
Stoma length	0.038–0.048	-	0.038–0.040	-
Procorpus length	0.300–0.340	-	0.283–0.320	-
Isthmus length	0.030–0.033	-	0.028–0.038	-
Diameter basal bulb	0.045–0.048	-	0.050–0.063	-
Esophagus length	0.390–0.420	-	0.378–0.400	-
Nerve ring-head end	0.150–0.168	-	0.133–0.158	-
Excretory pore-head end	0.480–0.560		0.430–0.490	
Cloaca-tail end	0.028–0.030	-	0.028–0.038	-
Median papilla-tail end	0.115–0.133	-	0.128–0.148	-
Dorso-lateral papilla-tail end	0.055–0.070	-	0.058–0.065	-
a	11.56–15.68	-	11.55–14.00	-
b	3.13–3.63	-	3.10–4.20	-
c	43.33–51.27	-	33.87–61.09	-

### Longior similis

Morffe, García & Ventosa, 2009

**Figure 4.**
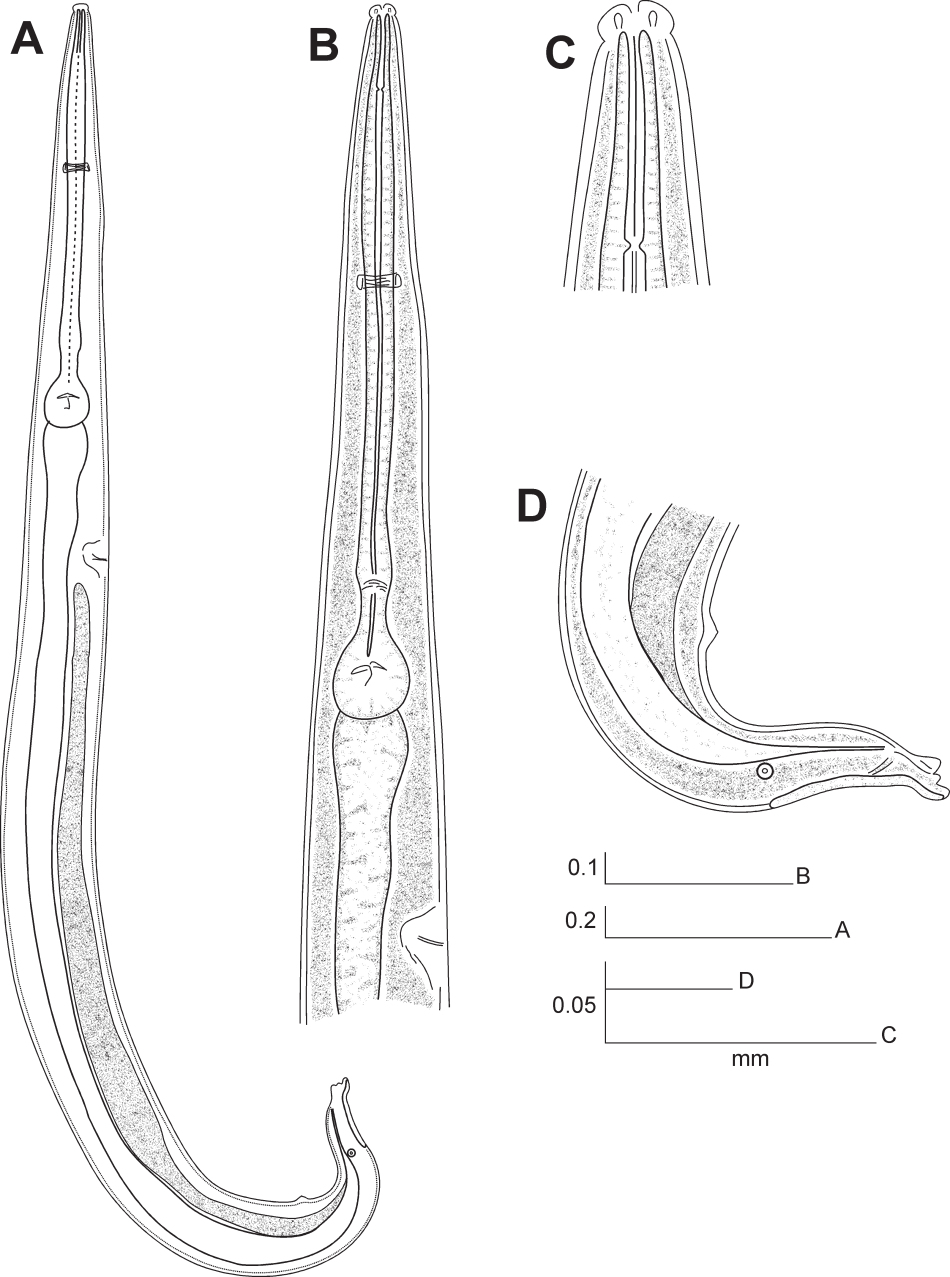
Longior similis Morffe, García & Ventosa, 2009 (male). **A** Entire nematode, lateral view **B** Esophageal region, lateral view **C** Cephalic end **D** Tail, lateral view.

**Figure 5.**
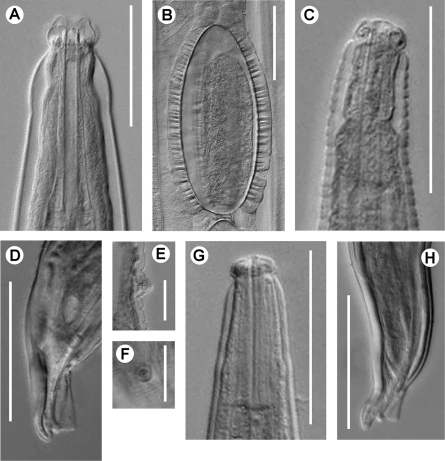
Longior longior Morffe & García sp. n. (female). **A** Cephalic end **B** Egg. Longior longior Morffe & García sp. n. (male) **C** Cephalic end **D** Tail, lateral view **E** Pre-cloacal median mammiform papilla, lateral view **F** Post-cloacal dorso-lateral papilla. Longior similis Morffe, García & Ventosa, 2009 (male) **G** Cephalic end **H** Tail, lateral view. Scale bars: A, B, C, D, G, H. 0.05 mm. E, F. 0.020 mm.

Figure 4 A–D Figure 5 G–H 

#### Material examined.

♂ voucher; Cuba, La Habana Province, Jaruco, Escaleras de Jaruco; in Passalus interstitialis; 16.III.2008; E. Fonseca, J. Morffe, F. Alvarez coll.; CZACC 11.4630.

#### Measurements.

Male (n = 1) a = 15.66, b = 4.15, c = 54.80, total length = 1.370, maximum body width = 0.088, stoma length = 0.045, procorpus length = 0.260, isthmus length = 0.028, diameter of basal bulb = 0.040, total length of oesophagus = 0.330, nerve ring to anterior end = 0.145, excretory pore to anterior end = 0.500, cloaca to posterior end = 0.025, distance from the mammiform papillae to the posterior end = 0.153, distance from the dorso-lateral papillae to the posterior end = 0.068.

#### Description.

Male body comparatively slender, shorter than female. Cuticle thin, finely annulated. Sub-cuticular longitudinal striae present. Head short, rounded, set-off from body by a single groove, papillae not observed. First cephalic annule not differentiated. Stoma as in the female. Oesophagus with its second half slender than first, hardly set-off from the isthmus. Basal bulb rounded, valve plate developed. Intestine simple, slender, its fore region dilated. Anus inconspicuous. Nerve ring encircling procorpus at about 40% of its length. Excretory pore located at about 1.5 body widths posterior to basal bulb. Testis single, full of spermatids, commencing just behind excretory pore, its second third slightly inflated. Spicule absent. A large, mid-ventral, precloacal, mammiform papilla situated at about 1.5 body widths before the tail tip. A pair of dorso-lateral, precloacal papillae at little less than a body width before the tail tip. Dorsal cuticle of the posterior end thickened from the level of the pair of papillae to almost the tail tip. Ventral cuticle expanded, forming a bursa-like structure with the distal end terminating before the tail tip. Tail short, conical, ending in a rounded tip. Posterior end slightly reflexed toward dorsal region.

#### Differential diagnosis (only based on males).

The male of Longior similis resembles Longior longicollis mainly by the form of the tail endbut differs by having the nerve ring displaced to the first half of the procorpus, at about the 40% of its length (in Longior longicollis the nerve ring is at the middle of procorpus). The procorpus of Longior longicollis is cylindrical in all its extension, while in Longior similis it is slightly wider in its first half. The tail is also comparatively shorter in the Cuban species (c = 54.8: 30.66–32.83).

From Longior longior sp. n., the other species where the male is known differs by having the cervical cuticle without visible annule and the tail tip reflexed toward the back. Longior longior sp. n. has the cervical cuticle annulated up to about the first third of the procorpus and the tail tip unreflexed. Also, Longior similis has a comparatively shorter oesophagus (b =4.15 *vs.* 3.13–3.63).

#### Host.

Passalus interstitialis Escholtz, 1829 (Coleoptera, Passalidae).

#### Site.

Gut caeca.

#### Locality.

Escaleras de Jaruco, Jaruco, La Habana Province, Cuba.

## Key to the species of Longior from Cuba

Note: males are described only in two of the five Cuban Longior species. Due to this, the present key was made only on the basis of females.

**Table:** 

1	Isthmus long and bent	Longior alius Coy & García, 1994
–	Ishmus short and not bent	2
2	First cephalic annule not inflated	Longior elieri García, Ventosa & Morffe, 2009
–	First cephalic annule more or less inflated	3
3	Cervical cuticle without wide annule, ovary reflected at about two body widths beyond the excretory pore	Longior longior sp. n. Morffe & García
–	Cervical cuticle with wide annule, ovary reflected at little less than a body width beyond the excretory pore	Longior similis Morffe, García & Ventosa, 2009

## Supplementary Material

XML Treatment for Longior

XML Treatment for Longior longior

XML Treatment for Longior similis
